# Cerebro-cerebellar functional neuroplasticity mediates the effect of electric field on electroconvulsive therapy outcomes

**DOI:** 10.1038/s41398-023-02312-w

**Published:** 2023-02-06

**Authors:** Zening Fu, Christopher C. Abbott, Jeremy Miller, Zhi-De Deng, Shawn M. McClintock, Mohammad S. E. Sendi, Jing Sui, Vince D. Calhoun

**Affiliations:** 1 Tri-Institutional Center for Translational Research in Neuroimaging and Data Science (TReNDS), Georgia State University, Georgia Institute of Technology, Emory University, Atlanta, GA USA; 2 Department of Psychiatry, University of New Mexico, Albuquerque, NM USA; 3 Computational Neurostimulation Research Program, Noninvasive Neuromodulation Unit, Experimental Therapeutics and Pathophysiology Branch, National Institute of Mental Health, National Institutes of Health, Bethesda, MD USA; 4 Department of Psychiatry and Behavioral Sciences, Duke University School of Medicine, Durham, NC USA; 5 Division of Psychology, Department of Psychiatry, UT Southwestern Medical Center, Dallas, TX USA; 6 Department of Psychiatry, Massachusetts General Hospital, Harvard Medical School, Charlestown, MA USA; 7 State Key Laboratory of Cognitive Neuroscience and Learning, Beijing Normal University, Beijing, China; 8 Department of Electrical and Computer Engineering, Georgia Institute of Technology, Atlanta, GA USA

**Keywords:** Neuroscience, Depression, Predictive markers

## Abstract

Electroconvulsive therapy (ECT) is the most effective treatment for severe depression and works by applying an electric current through the brain. The applied current generates an electric field (E-field) and seizure activity, changing the brain’s functional organization. The E-field, which is determined by electrode placement (right unilateral or bitemporal) and pulse amplitude (600, 700, or 800 milliamperes), is associated with the ECT response. However, the neural mechanisms underlying the relationship between E-field, functional brain changes, and clinical outcomes of ECT are not well understood. Here, we investigated the relationships between whole-brain E-field (E_brain_, the 90^th^ percentile of E-field magnitude in the brain), cerebro-cerebellar functional network connectivity (FNC), and clinical outcomes (cognitive performance and depression severity). A fully automated independent component analysis framework determined the FNC between the cerebro-cerebellar networks. We found a linear relationship between E_brain_ and cognitive outcomes. The mediation analysis showed that the cerebellum to middle occipital gyrus (MOG)/posterior cingulate cortex (PCC) FNC mediated the effects of E_brain_ on cognitive performance. In addition, there is a mediation effect through the cerebellum to parietal lobule FNC between E_brain_ and antidepressant outcomes. The pair-wise t-tests further demonstrated that a larger E_brain_ was associated with increased FNC between cerebellum and MOG and decreased FNC between cerebellum and PCC, which were linked with decreased cognitive performance. This study implies that an optimal E-field balancing the antidepressant and cognitive outcomes should be considered in relation to cerebro-cerebellar functional neuroplasticity.

## Introduction

Major depressive disorder (MDD) affects more than 163 million people, approximately 2% of the world’s population in 2017 [1]. MDD is characterized by persistently depressed mood, anhedonia, impaired cognitive function, and suicidal thoughts [2]. Electroconvulsive therapy (ECT) is one of the most effective treatments along with accelerated transcranial magnetic stimulation, repetitive transcranial magnetic stimulation, Ketamine, and deep brain stimulation for treatment-resistant depressive episodes, which passes a controlled electric current through the brain under general anesthesia, producing substantial improvement in 60 to 80 percent of patients [3]. Despite its effectiveness, ECT may cause cognitive side-effects, including impairment in attention, memory, and executive functioning [4–6]. ECT’s mechanisms of cognitive side-effects and antidepressant response are poorly understood. This gap in knowledge limits parameter development to optimize antidepressant benefits and reduce cognitive risk.

ECT promotes changes in how brain cells communicate to normalize aberrant depression-related brain functioning, which is commonly known as neuroplasticity [7]. A wide range of ECT-induced functional connectivity (the strength with which activity in brain regions correlates over time) changes have been implicated in both antidepressant and cognitive outcomes [8, 9]. ECT can normalize dysregulated brain networks in MDD [10], such as the default-mode network, which is involved in self-referential processing including emotion perception [11, 12]. A longitudinal study has demonstrated that ECT modulates the function of the default-mode network, accompanied by improved mood and impaired cognitive function, where the connectivity changes and impaired cognitive function recovered one month after the completion of ECT [13].

Besides these networks, the cerebellum has been recently investigated in neuroimaging studies of MDD and ECT. Conventionally regarded as a pure motor-control system, the cerebellum now is thought to play a relevant role in cognitive and emotional processing [14–16]. Mounting evidence has demonstrated that MDD patients exhibit cerebellum dysrhythmia structurally [17, 18] and functionally [19]. The cerebellum is considered in the pathological model of MDD and the alterations of cerebro-cerebellar connectivity implied neural deficits in depression [20]. Neuroimaging studies have also identified significant cerebellar changes following ECT [21, 22], indicating a potential association between ECT and cerebellar neuroplasticity. Cerebro-cerebellar connectivity changes may be associated with cognitive performance, which implies a potential neural pathway for the mitigation of ECT-induced side-effects [23]. Although increasing evidence has linked the functional connectivity changes to ECT, the mechanisms underlying the relationships between functional neuroplasticity and ECT response, especially the short-term cognition changes, are still unknown.

Research on electric field (E-field) modeling and ECT has tried to link the E-field strength to brain neuroplasticity, with robust associations identified between E-field and structural neuroplasticity [24]. Another study reported that E-field in the temporal lobes is correlated with less optimal ECT outcome [25]. In the context of E-field modeling, the electrode placement determines the geometric shape of the E-field, and the amplitude determines the E-field magnitude [26]. Note that the whole-brain E-field and stimulus amplitude is related (*r* = 0.7129, *p* = 6.31 × 10^−9^). However, with a fixed extracranial amplitude, the ECT “doses” as represented by the intracranial E-field is highly variable due to the anatomic difference in skin, skull, fluid, and brain tissue [27]. The anatomic variability is prominent in older patients with depressive episodes, which can compromise both antidepressant efficacy and safety. E-field is a more accurate depiction of the electric field dose relative to pulse amplitude. It requires pre-ECT anatomic images to achieve the goal of individualized amplitude and reducing the variability of ECT dose, equipment, and expertize. We believe that the investigation of E-field variability will create a more standardized and consistent ECT dosing strategy for treatment with ECT, thus improving the ECT-induced outcomes. We have previously identified a trade-off between amplitude strength on the antidepressant (higher is better) and cognitive outcomes (lower is better) [28]. We also demonstrated that hippocampal neuroplasticity significantly mediated the relationship between E-field strength and antidepressant outcomes, E-field strength was directly related to cognitive side-effects [29]. Nevertheless, previous ECT E-field investigations limited analyses to the structural changes induced by ECT. Functional neuroplasticity is also a key element in ECT investigations, as it may reflect the brain’s capability to restructure itself by forming new neural connections. To date, no study has examined the relationship between E-field strength, functional neuroplasticity, and clinical outcomes.

In this work, we shift the focus from structural neuroplasticity to functional neuroplasticity, and from localized analysis to whole-brain analysis. Via the fully automated independent component analysis (ICA) framework [30], we investigated cerebro-cerebellar functional connectivity neuroplasticity and its relationships with E-field strength, antidepressant outcomes, and cognitive side-effects. Antidepressant outcomes were measured by the Hamilton Depression Rating Scale-24 item (HDRS_24_) and cognitive side-effects were measured by Delis Kaplan Executive Function System (DKEFS) Verbal Fluency Test. We hypothesized that functional connectivity neuroplasticity mediates the effects of E-field strength on both antidepressant outcomes and cognitive side-effects.

## Materials and methods

### Study design and participants

This study was approved by the University of New Mexico (UNM) Human Research Protection office (clinical trial registration number: NCT02999269). Procedural consent or assented to the study participation was obtained from all participants under protocols approved by the Institutional Review Board of UNM. 62 participants with a diagnosis of MDD and ages ranging between 50 to 80 years old were recruited in this investigation. Subjects have failed multiple appropriate medication trials and met clinical indications for ECT.

**Fig. 1:**
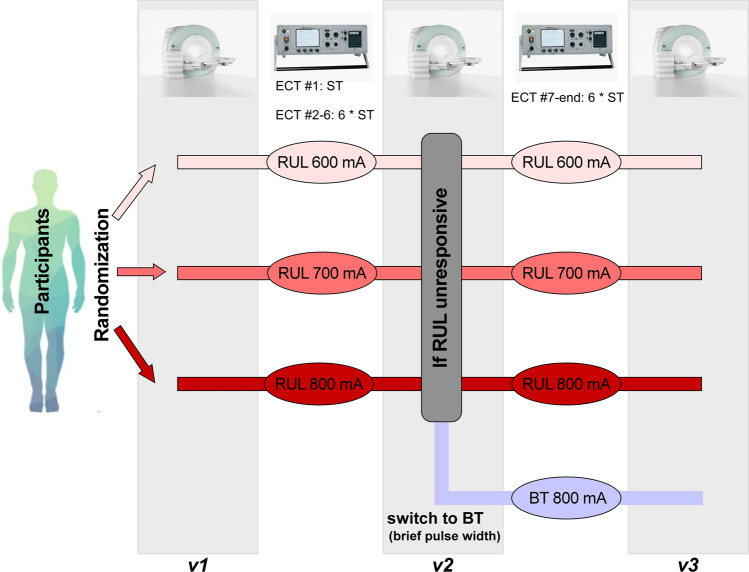
Study design of the ECT data. Participants were randomized to 600, 700, and 800 mA pulse amplitudes and started the ECT series with RUL electrode placement. Participants received antidepressant ratings, neuropsychological assessments, and imaging sessions pre-ECT (v1), mid-ECT (v2), and post-ECT (v3). If participants had <25% reduction in v2 HDRS (compared to v1), they were switched to BT electrode placement with brief pulse width. *ECT* electroconvulsive therapy, *mA* milliamperes, *RUL* right unilateral, *HDRS* Hamilton Depression Rating Scale-24 item, *BT* bitemporal.

Prior to the baseline ECT assessment, medication tapering was scheduled for every participant with only as-needed medications permissible. This double-blind investigation (raters and subjects were blinded to randomization) randomized subjects to three pulse amplitude (600, 700, or 800 milliamperes [mA]) [28, 31], starting the ECT treatment with right unilateral (RUL) electrode placement. An ultra-brief pulse width (0.3 milliseconds [ms]) was used in the ECT series initially. 15 subjects switched to brief (1.0 ms) pulse width because of the reduced antidepressant outcomes in the lower amplitude arms. The primary antidepressant outcome measure in this investigation is the Hamilton Depression Rating Scale-24 item (HDRS_24_) and the cognitive battery measure is the Delis Kaplan Executive Function System (DKEFS) Verbal Fluency Test [32, 33]. DKEFS Verbal Fluency Test is a valid measure of cognitive side-effects because it is sensitive to the detection of amplitude-mediated neurocognitive impairment [28] and has been associated with structural neuroplasticity [29]. Subjects blinded to treatment-arm assignment received clinical, cognitive, and imaging assessments Pre-ECT (v1, before ECT treatments), mid-ECT (v2, after the six ECT treatments), and post-ECT (v3, within one week of finishing all ECT series) [28, 34]. If subjects were nonresponsive to the assigned ECT treatments, they then switched to bitemporal (BT) electrode placement with a brief (1.0 ms) pulse width for the remaining ECT series (Fig. 1). The ECT imaging (unprocessed), clinical, and demographic data have been transferred to the National Data Archive. More details of the study design, participants, and clinical and cognitive assessments can be found in [28] and the supplementary materials.

### Image acquisition and preprocessing

Resting-state fMRI, T1, and T2 data were collected by a 3T-Siemens scanner. Each subject can have one or two resting-state fMRI scans for each session (v1–v3), and the total acquisition time for each scan is 5:09 (minutes: seconds). We preprocessed the resting-state fMRI data using a combination of the FMRIB Software Library v6.0 (FSL) toolbox and the Statistical Parametric Mapping 12 (SPM 12) toolbox, under the MATLAB 2019b environment. The preprocessing steps include distortion correction, slice timing correction, head motion realignment, normalization to the standard Montreal Neurological Institute (MNI) space, and smoothing. Details of image acquisition and preprocessing are provided in the supplementary materials.

We performed quality control (QC) on the preprocessed resting-state fMRI to select subjects for further analysis. We excluded subjects’ scans if their head motions were larger than 3 mm or 3°. Additionally, we excluded subjects who did not show good normalization of their fMRI images to the MNI standard space (by comparing the individual mask with the group mask). Details of the subject inclusion criteria are provided in the supplementary materials.

### E-Field modeling

The Simulation of Non-Invasive Brain Stimulation (SimNIBS) toolbox was used for E-field modeling to generate a subject-specific anatomically realistic volume conductor model [35]. Via a combination of the FSL toolbox and the SPM 12 toolbox, T1- and T2-weighted images were segmented into skin, bone, eyes, cerebral spinal fluid, ventricles, and gray and white matter. The segmented tissue compartments were meshed into a head model using Gmsh, and ECT electrodes were added to the head mesh in either RUL or received BT configuration, stimulated with 600, 700, or 800 mA as per arm assignment. The voltages and electric fields that correspond to the stimulation configuration were calculated throughout the head mesh.

Based on the electrode placement (BT or RUL) and the amplitude (600, 700, or 800 mA) from the last treatment of the ECT series, we calculated the whole-brain E-field strength (E_brain_). E_brain_ was measured as the 90^th^ percentile of E-field magnitude across the whole brain. E_brain_ at 90^th^ percentile is standard based on previous E-field investigations [29, 36]. E_brain_ at 90^th^ percentile is strongly correlated with those calculated at other percentiles: 50^th^ (*r* = 0.95), 75^th^ (*r* = 0.99), 85^th^ (*r* = 1.0), and 95^th^ (*r* = 1.0).

### Neuromark framework and functional network connectivity

The QC resting-state fMRI data were analyzed via the Neuromark framework which provides a robust estimation of functional networks across subjects [30]. Neuromark is a unified ICA framework that adopts two large healthy datasets to construct a group of replicable networks as references. Meaningful networks were labeled as intrinsic connectivity networks (ICN) by inspecting the locations of the peak activations of their spatial maps and the low-frequency fluctuations of their time courses (TCs). By using these ICN templates in a spatial-constrained ICA, Neuromark can extract comparable functional networks and their TCs in new data. This framework leverages the advantages of a data-driven approach that retains more variability specific to each data and provides comparability across subjects, sessions, and scans that benefit our statistical analysis. The effectiveness of Neuromark has been demonstrated in previous studies, with numerous brain network abnormalities identified across datasets and brain disorders [37–40].

TCs of ICNs from each scan underwent additional post-processing to remove the remaining noise. These procedures included: (1) detrending linear, quadratic, and cubic trends; (2) removal of detected outliers; and (3) band-pass filtering with a cutoff frequency of 0.01–0.15 Hz. We then calculated the Pearson correlation coefficient between cerebellar ICNs and cerebral ICNs to measure cerebro-cerebellar functional network connectivity (FNC). Note that, if there are two scans available for a session, we averaged the FNC across two scans and used the mean FNC for the investigation. The Neuromark framework is implemented in the group ICA of the fMRI toolbox (GIFT; http://trendscenter.org/software/gift) and more details of Neuromark are provided in the supplementary materials.

### Statistical analysis

#### E_brain_, antidepressant outcomes and cognitive side-effects

A general linear model (GLM) was used to investigate the relationship between E_brain_ and the antidepressant outcome, which was measured by the percent change in HDRS total score (v1–v3) relative to pre-ECT HDRS total score (ΔHDRS[%]). Age, gender, pulse width, and treatment number were set as the covariates in the GLM. We also used GLM to investigate the relationship between E_brain_ and cognitive side-effects, controlling for gender, pulse width, treatment number, and intelligence (measured by the Test of Premorbid Functioning Score [TOPF]). TOPF is a measure of premorbid intelligence and we controlled for premorbid intelligence because it has been shown that subjects with higher premorbid intelligence can better compensate for the impact of ECT on cognitive functions [41, 42]. The cognitive outcomes were measured by the changes in DKEFS Verbal Fluency Test scaled score between v1 and v3 (ΔDKEFS Verbal Fluency Test). Electrode placement influences E_brain_ through E-field geometry and was not included as a covariate. Age was accounted for in the DKEFS Verbal Fluency Test scaled score and therefore not included as a covariate in the cognitive analyses [29]. We calculated the effect size of each analysis using partial eta-squared η^2^p. η^2^p was calculated as $\eta ^2p = \frac{{SS_{eff}}}{{SS_{eff} + SS_{res}}}$, where *SS*_*eff*_ is the sum of squares of the effect and *SS*_*res*_ is the sum of squares of the residual.

#### FNC neuroplasticity, E_brain_ and clinical outcomes

We first pre-selected cerebro-cerebellar FNC pairs whose changes are related to E_brain_. A GLM was used to investigate the relationship between E_brain_ and FNC changes between v1 and v3 (FNC_v1_- FNC_v3_). Age, gender, pulse width, and treatment number were the covariates controlled in the GLM analysis. The FNC pairs with significance *p* ≤ 0.05 were selected for further analysis of associations between FNC changes and clinical outcomes. Then we assessed the relationships between FNC changes and antidepressant outcomes (ΔHDRS), controlling for age, gender, pulse width, and treatment number using GLM. In parallel, we examined the relationships between FNC changes and cognitive side-effects (ΔDKEFS Verbal Fluency Test), controlling for gender, pulse width, treatment number, and TOPF. The resulting associations between FNC and clinical outcomes are corrected by FDR correction (p < 0.05).

#### FNC neuroplasticity mediates the relationships between E_brain_ and clinical outcomes

A standard mediation analysis was performed using the Mediation Toolbox (https://github.com/canlab/MediationToolbox). This toolbox has been successfully applied in many neuroimaging studies, with numerous mediated effects identified between imaging features and clinical assessments [43–45]. In this study, we used a standard three-variable path model, with E_brain_ as the independent variable, ∆DKEFS as the dependent variable, and FNC changes as the mediator. Confounding variables as in the association analysis (gender, pulse width, treatment number, and intelligence [TOPF]) were regressed out in the mediation model. We hypothesized that the FNC changes mediate the relationships between E_brain_ and ECT-induced side-effects. Likewise, we investigated the mediation effects of FNC changes on the association between E_brain_ and ∆HDRS. Age, gender, pulse width, and treatment number were set as the confounding variables in the mediation model. The significance of the mediation was estimated by the bias-corrected bootstrap approach with 10000 random samplings.

#### E_brain_-FNC neuroplasticity-cognition pathway

We further performed pair-wise t-tests to examine how E_brain_ influences FNC changes and then cognitive side-effects. We first divided the subjects into two groups according to their E_brain_ (group 1 with E_brain_ < mean[E_brain_] and group 2 with E_brain_ > mean[E_brain_]). Pair-wise t-tests were performed for each group to investigate whether their FNC changed significantly following ECT (between v1 and v3). Then we divided the subjects into another two groups according to the changes in their FNC (group 1 with ∆FNC < 0 and group 2 with ∆FNC > 0). We performed pair-wise t-tests for all subjects as well as each group to investigate whether subjects with different FNC alterations show significant cognitive impairment after ECT.

## Results

### Brain parcellation

**Fig. 2:**
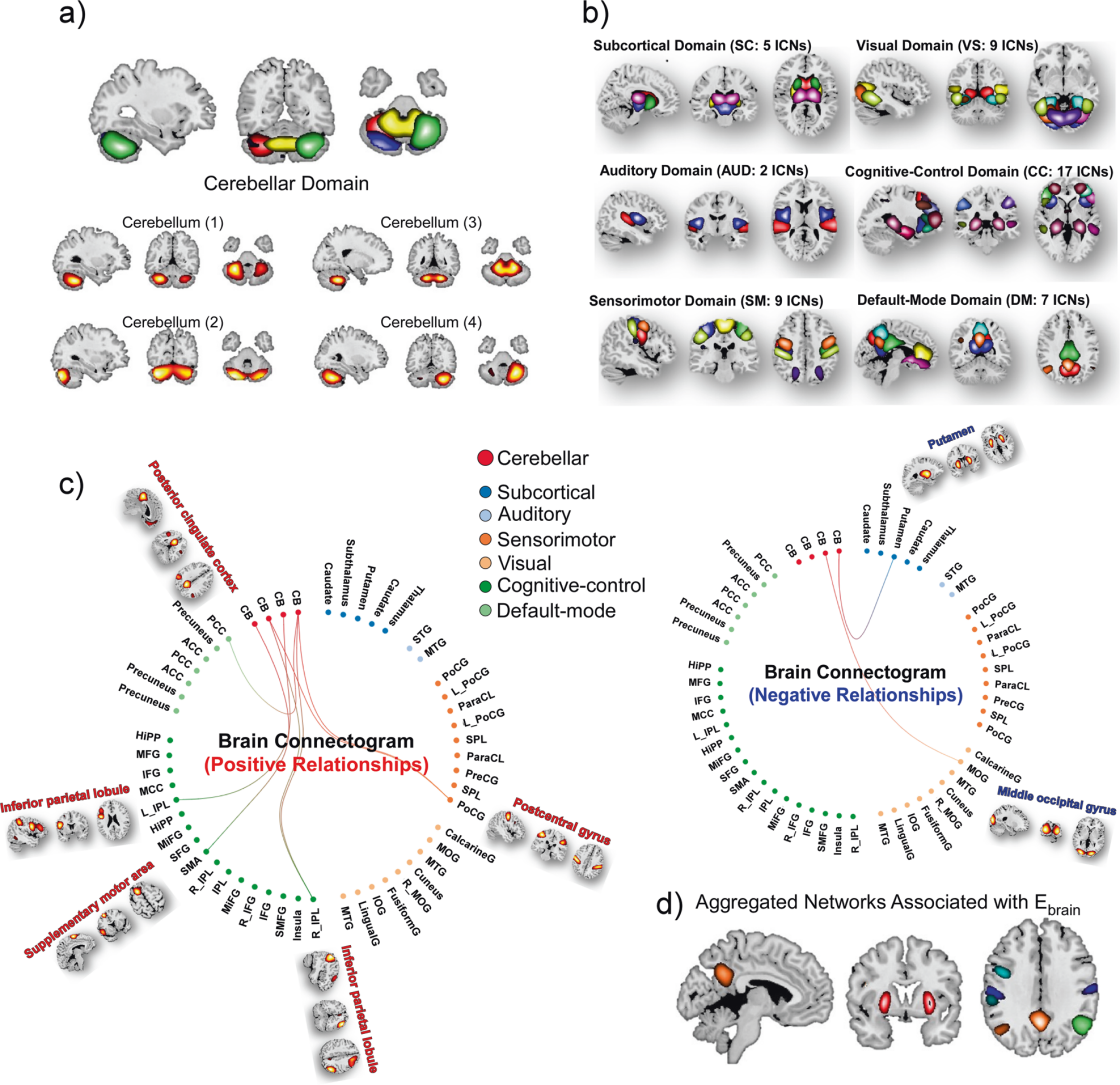
Spatial maps of ICNs and E_brain_ related cerebro-cerebellar FNC. **a**, **b** Four cerebellar and 49 cerebral networks are identified via the Neuromark framework, arranging into CB, SC, AUD, SM, VS, CC, and DM domains. **c**, **d** Functional connectogram of FNC with changes significantly associated with E_brain_ (*p* < 0.05). **e** spatial maps of aggregated cerebral networks that with cerebro-cerebellar FNC related to E_brain_. *ICN* intrinsic connectivity network, *E*_*brain*_ the 90^th^ percentile of electric field magnitude in the brain, *FNC* functional network connectivity, *CB* cerebellar domain, *SC* subcortical domain, *AUD* auditory domain, *SM* sensorimotor domain, *VS* visual domain, *CC* cognitive-control domain, *DM* default-mode domain.

In total 50 subjects were selected in the present study and detailed demographic information is provided in Table S1. Subjects have at least one good resting-state fMRI scan at both v1 and v3 sessions that passed the Neuromark QC. Assuming moderate observational errors (~5% of the mean) in neuroimaging data and weak to moderate effects (~15% of the variability in X explained by Y), our sample size of *n* = 50 subjects is adequate to achieve power equal to 0.80 and a Type I error equal to 0.05 in the correlation analysis. The brain was parcellated into 53 meaningful components, namely ICNs, using the Neuromark framework [30]. The 53 ICNs include four cerebellar networks assigned to the cerebellar domain (CB) and 49 cerebral networks assigned to six functional domains: subcortical (SC), auditory (AUD), visual (VS), sensorimotor (SM), cognitive-control (CC), and default-mode (DM) domains. The spatial maps of ICNs from the cerebellar and cerebral domains are displayed in Fig. 2a, b, respectively. Details of the ICN coordinates and labels are provided in the supplementary materials.

### E_brain_ related cerebro-cerebellar functional network connectivity neuroplasticity

Cerebro-cerebellar FNC was measured by the Pearson correlation coefficient between TCs of ICNs. There are nine cerebro-cerebellar FNC pairs whose changes are correlated with E_brain_ (*p* < 0.05, uncorrected, Fig. 2c). Specifically, ∆FNC between putamen/middle occipital gyrus (MOG) and cerebellum are negatively correlated with E_brain_. ∆FNC between postcentral gyrus/inferior parietal lobule (IPL)/supplementary motor area (SMA)/posterior cingulate cortex (PCC) and cerebellum are positively correlated with E_brain_ (Fig. 2d).

### ∆Cerebro-cerebellar FNC mediate effects of E_brain_ on cognitive side-effects

**Fig. 3:**
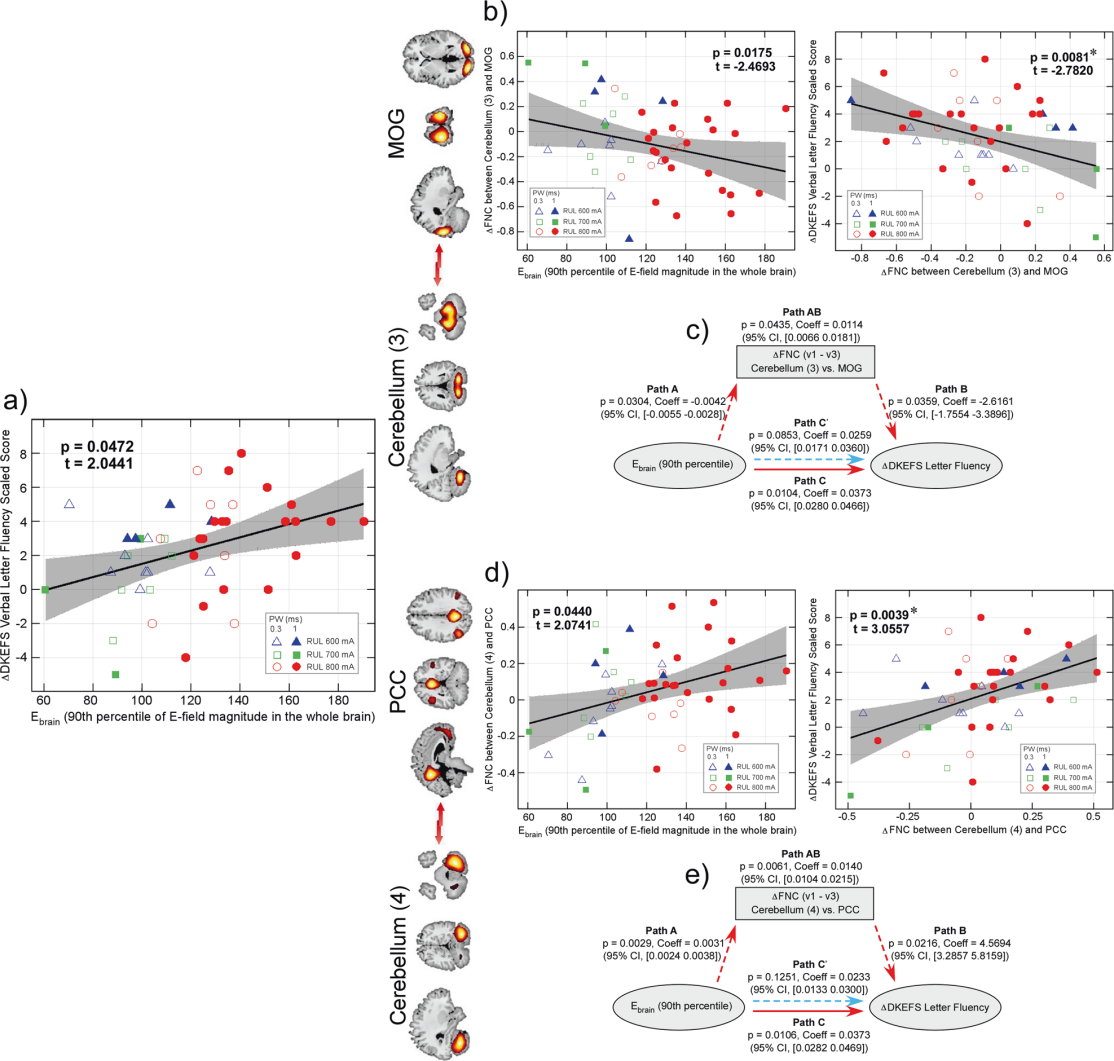
E_brain_, FNC neuroplasticity and cognitive side-effects. **a** E_brain_ is positively correlated with the change in DKEFS Verbal Fluency - Letter Fluency. **b** Changes in FNC between cerebellum (3) and MOG is negatively correlated with E_brain_ and the change in DKEFS Verbal Fluency - Letter Fluency. **c** ∆cerebellum-MOG FNC mediates the effects of E_brain_ on ∆DKEFS Verbal Fluency - Letter Fluency. **d** Changes in FNC between cerebellum (4) and PCC is positively correlated with E_brain_ and the change in DKEFS Verbal Fluency - Letter Fluency. **e** ∆cerebellum-PCC FNC mediates the effects of E_brain_ on ∆DKEFS Verbal Fluency - Letter Fluency. * Significance *p* < 0.05, FDR corrected. *E*_*brain*_ the 90^th^ percentile of electric field magnitude in the brain, *FNC* functional network connectivity, *DKEFS* Delis Kaplan Executive Function System, *MOG* middle occipital gyrus, *PCC* posterior cingulate cortex, *FDR* false discovery rate.

E_brain_ is positively correlated with the changes in DKEFS Verbal Fluency - Letter Fluency scaled score (*p* = 0.0472, t = 2.0441, beta = 0.0338, effect size = 0.1209, Fig. 3a), indicating that larger E_brain_ is associated with larger impairment in cognitive performance (v1–v3). Among the nine E_brain_ related FNC pairs, change of FNC between cerebellum (3) and MOG is negatively correlated with change in DKEFS Verbal Fluency - Letter Fluency (*p* = 0.0081, false discovery rate [FDR] corrected across nine pairs, t = −2.7802, beta = −3.1879, effect size = 0.1582, Fig. 3b). The mediation analysis shows that the changes of cerebellum-MOG FNC significantly mediate the effect of E_brain_ on ∆DKEFS Verbal Fluency - Letter Fluency. While the direct effect of E_brain_ on ∆DKEFS Verbal Fluency - Letter Fluency is insignificant (Path C’: *p* > 0.05), there are significant indirect effects through ∆cerebellum-MOG FNC (Path A: *p* = 0.0304, Coeff = −0.0042, CI, −0.0055~−0.0028; Path B: *p* = 0.0359, Coeff = −2.6161, CI, −1.7554~−3.3896; Path AB: *p* = 0.0435, Coeff = 0.0114, CI, 0.0066~0.0181), resulting in a significant total effect (Path C: *p* < 0.0104, Coeff = 0.0373, CI, 0.0280~0.0466, Fig. 3c).

In addition, change of FNC between cerebellum (4) and PCC (one of the nine E_brain_ related FNC pairs) is positively correlated with change in DKEFS Verbal Fluency - Letter Fluency (*p* = 0.0039, FDR corrected, t = 3.0557, beta = 5.3953, effect size = 0.1956, Fig. 3d). Similarly, the mediation analysis shows that while the direct effect of E_brain_ on ∆DKEFS Verbal Fluency - Letter Fluency is insignificant (Path C’: *p* > 0.05), there are significant indirect effects through ∆cerebellum-PCC FNC (Path A: *p* = 0.0029, Coeff = 0.0031, CI, 0.0024~0.0038; Path B: *p* = 0.0216, Coeff = 4.5694, CI, 3.2875~5.8159; Path AB: *p* = 0.0061, Coeff = 0.0140, CI, 0.0104~0.0215), resulting in a significant total effect (Path C: *p* < 0.0106, Coeff = 0.0373, CI, 0.0282–0.0469, Fig. 3e).

### ∆Cerebro-cerebellar FNC mediate effects of E_brain_ on antidepressant outcomes

**Fig. 4:**
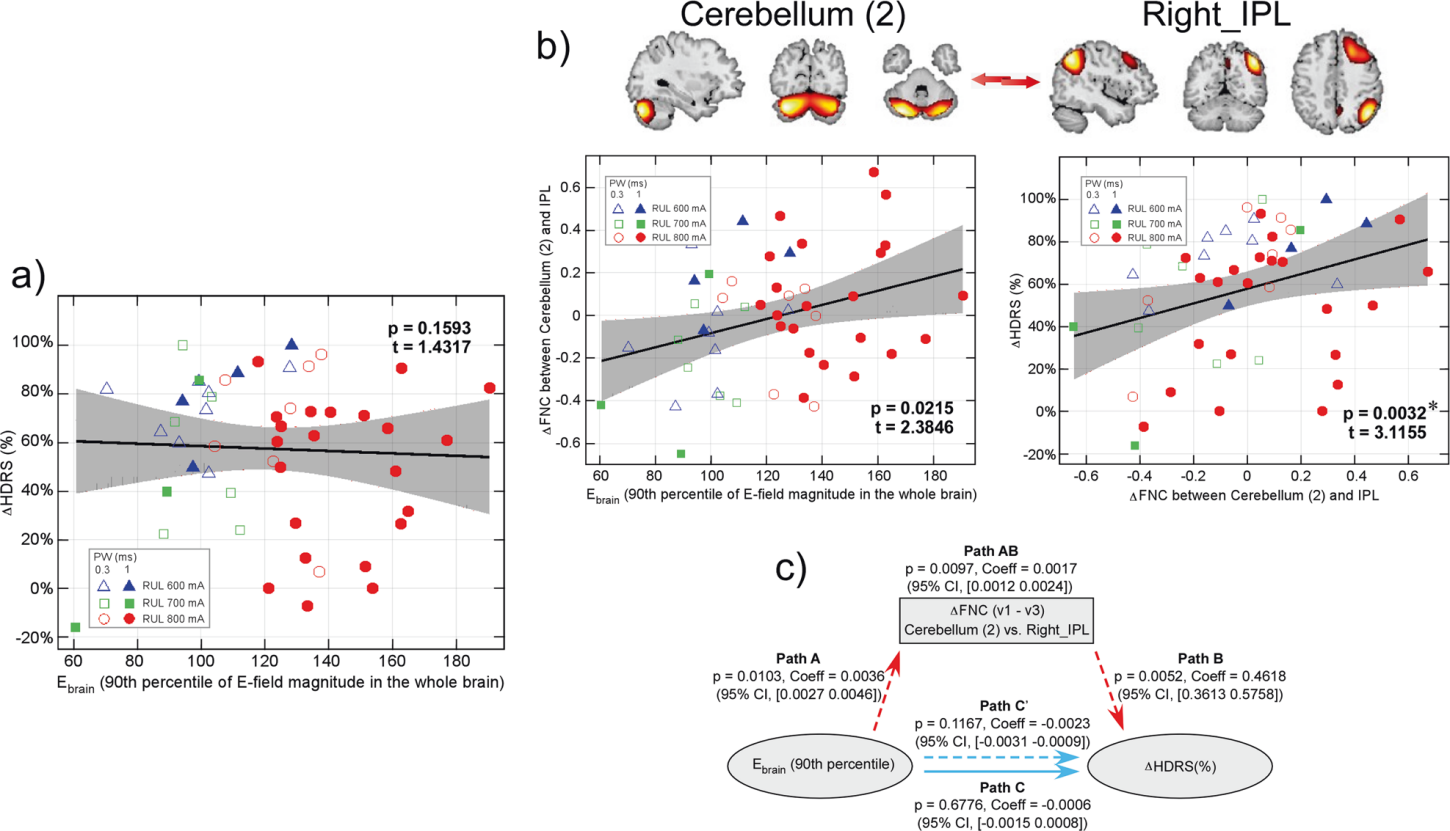
E_brain_, cerebellum-right IPL FNC neuroplasticity and antidepressant outcomes. **a** E_brain_ is not correlated with ∆HDRS. **b** changes in FNC between cerebellum (2) and right IPL is positively correlated with E_brain_ and ∆HDRS. **c** ∆cerebellum-IPL FNC mediates the effects of E_brain_ on ∆HDRS. * Significance *p* < 0.05, FDR corrected. *E*_*brain*_ the 90^th^ percentile of electric field magnitude in the brain, *IPL* inferior parietal lobule, *FNC* functional network connectivity, *HDRS* Hamilton Depression Rating Scale-24 item, *FDR* false discovery rate.

E_brain_ has no relationships with ∆HDRS (%) (*p* = 0.1593, t = 1.4317, beta = 0.0027, effect size = 0.0671, Fig. 4a). Although there is no relationship between E_brain_ and ∆HDRS, change in FNC between cerebellum (2) and right IPL (one of the nine E_brain_ related FNC pairs) is positively correlated with ∆HDRS (*p* = 0.0032, FDR corrected, t = 3.1155, beta = 0.4544, effect size = 0.2025, Fig. 4b). The mediation analysis shows that the changes of cerebellum-IPL FNC significantly mediate the effect of E_brain_ on ∆HDRS. There are significant indirect effects between E_brain_ and ∆HDRS through ∆cerebellum-IPL FNC (Path A: *p* = 0.0103, Coeff = 0.0036, CI, 0.0027–0.0046; Path B: *p* = 0.0052, Coeff = 0.4618, CI, 0.3613–0.5758; Path AB: *p* = 0.0097, Coeff = 0.0017, CI, 0.0012–0.0024). The direct effect of E_brain_ on ∆HDRS (although it is a weak trend: *p* = 0.1167) is inverse to the indirect effect, which leads to an insignificant total effect of E_brain_ on ∆HDRS (*p* > 0.05).

### E_brain_ related FNC neuroplasticity predict cognitive performance

**Fig. 5:**
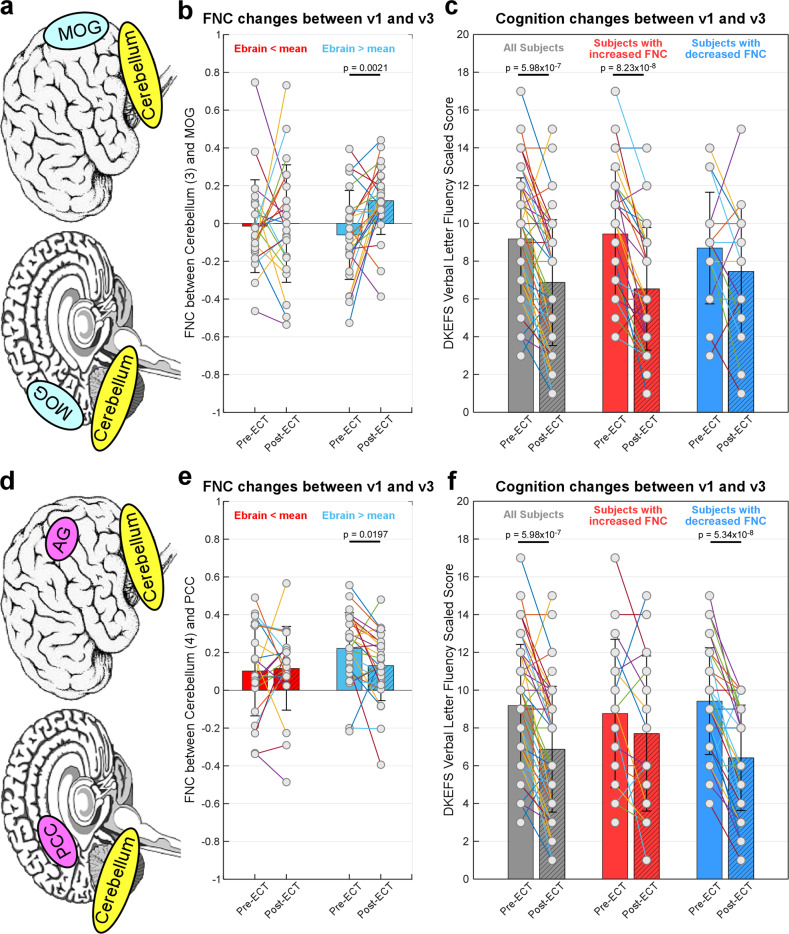
E_brain_ related cerebro-cerebellar FNC neuroplasticity can predict ECT induced cognitive impairment. **a** ∆FNC between cerebellum and MOG is associated with E_brain_ and cognitive side-effects. **b** Subjects with large E_brain_ tend to have increased cerebellum-MOG FNC. **c** Increased cerebellum-MOG FNC results in a decrease of DKEFS Verbal Fluency - Letter Fluency. **d** ∆FNC between cerebellum and PCC is associated with E_brain_ and cognitive side-effects. **e** Subjects with large E_brain_ tend to have decreased cerebellum-PCC FNC. **f** Decreased cerebellum-PCC FNC results in a decrease of DKEFS Verbal Fluency - Letter Fluency. *E*_*brain*_ the 90^th^ percentile of electric field magnitude in the brain, *FNC* functional network connectivity, *ECT* electroconvulsive therapy, *MOG* middle occipital gyrus, *DKEFS* Delis Kaplan Executive Function System, *PCC* posterior cingulate cortex.

The results of the pair-wise t-test show that subjects with larger E_brain_ (> mean[E_brain_]) tend to have increased FNC between the cerebellum and MOG (*p* = 0.0021, t = −3.4293, Fig. 5b), while subjects with smaller E_brain_ (< mean[E_brain_]) do not show significant changes in this FNC pair (*p* > 0.05, Fig. 5b). Significant cognitive impairment after ECT can be observed by using all samples in the statistical analysis (*p* = 5.98 × 10^−7^, *t* = 5.7704, Fig. 5c). Interestingly, we found that only those subjects with increased FNC between the cerebellum and MOG (∆FNC_v1-v3_ < 0) show a significant cognitive impairment after ECT (*p* = 8.23 × 10^−8^, t = 7.0278, Fig. 5c), while subjects without increased FNC do not show this cognitive impairment (*p* > 0.05, Fig. 5c). Our findings suggest that although a significant cognitive impairment after ECT can be observed at the whole group level, subjects with different FNC changes might have different levels of cognitive impairment.

Our results also demonstrate that subjects with larger E_brain_ (>mean[E_brain_]) tend to have decreased FNC between the cerebellum and PCC (*p* = 0.0197, t = 2.4924, Fig. 5e), while subjects with smaller E_brain_ (<mean[E_brain_]) do not show significant changes in this FNC pair (*p* > 0.05, Fig. 5e). Similarly, we found that only those subjects with decreased FNC between the cerebellum and PCC (∆FNC_v1-v3_ > 0) show a significant cognitive impairment after ECT (*p* = 5.34 × 10^−8^, t = 7.1880, Fig. 5f), while the subjects without decreased cerebellum-PCC FNC do not show this cognitive impairment (*p* > 0.05, Fig. 5f).

It should be noted that, in the above correlation and mediation analyses, both FNC change and DKEFS change were calculated as the change between v1 and v3 (v1–v3), where a positive value indicates decreased FNC/DKEFS after ECT while a negative value indicates increased FNC/DKEFS after ECT. The t-test results are consistent with the above findings by showing that high E_brain_ results in an increase in the FNC between the cerebellum and MOG and a decrease in the FNC between the cerebellum and PCC, which might be associated with more cognitive impairment.

We also performed similar t-test analyses (as those in the section “*E*_*brain*_*-FNC neuroplasticity-cognition pathway*”) for the HDRS and provided the results in the supplementary materials. Our findings support the results of the mediation analysis, where lower E_brain_ is associated with decreased FNC, which might result in fewer antidepressant outcomes.

## Discussion

This study provides new insights into the relationships between E-field strength, functional neuroplasticity, and clinical response to ECT. The key findings include changes in cerebro-cerebellar functional connectivity in association with the E-field. Specifically, two cerebro-cerebellar FNC decrease, and seven cerebro-cerebellar FNC increase as E-field strength increases. Further, the E_brain_-related cerebro-cerebellar FNC is associated with both antidepressant outcomes and cognitive side-effects following ECT. Functional neuroplasticity through cerebro-cerebellar FNC mediates the effects of E_brain_ on both antidepressant outcomes and cognitive side-effects. Our results demonstrate that E-field strength is associated with ECT-mediated cognitive impairment, specifically verbal dysfluency, through increased FNC between the cerebellum and MOG and decreased FNC between the cerebellum and PCC. E-field strength is also associated with antidepressant outcomes through increased FNC between the cerebellum and IPL.

Mechanisms of ECT-related neuroplasticity, including neurogenesis, angiogenesis, synaptogenesis, and gliogenesis may be associated with the changing electric field [7]. The neurogenic hypothesis posits that the depressive brain has an impairment of producing new neurons for proper mood control, and generating new neurons is beneficial for antidepressant efficacy [46, 47]. However, it remains heavily debated [48, 49] that neurogenesis cannot be supported by existing ECT-imaging literature given the short time frame of the ECT series and widespread structural neuroplasticity [50–54]. Our results reveal potential relationships between E-field strength and functional neuroplasticity (*p* < 0.05, uncorrected), though their correlations are not significant after the multiple comparison correction. We speculate that the increased brain volumes might change the capabilities and behaviors of neurons to communicate, as reflected by the alterations of functional connectivity between brain regions [24]. Synaptogenesis and functional remodeling are possible mechanisms that may be compatible with both structural and functional neuroplasticity [55].

Using individualized connectomes, previous studies have found a set of functional connectivity patterns affected by ECT, whose changes directly or indirectly impact cognitive performance [9, 23]. These changes in connectivity involve a variety of functional networks, including the cognitive-control network, the default-mode network, and the cerebellum network [9, 56]. Interestingly, both increased and decreased cerebro-cerebellar connectivity have been observed after ECT, indicating the heterogeneous patterns of ECT modulated functional connectivity changes. The changed temporal coherence of functional connectivity results may be suggestive of synaptic remodeling, in which E-field affects the neurons to modulate the information flow by adopting polarized morphologies [57, 58].

In our study, cognitive outcomes were measured by the DKEFS Verbal Fluency Test, with a primary focus on letter fluency (e.g., phonemic fluency) [59]. The cerebellum shows a robust connection to many cognitive and affective cerebral structures [60], especially the default-mode network, a system responsible for self-referential information processing and memory [9]. Consistent with these findings, our results on the association between cognitive performance and decreased PCC-cerebellum connectivity provide further evidence that the default-mode network to cerebellum connectivity might play a direct role in regulating cognitive function [61, 62]. The biological underpinnings of ECT-induced cognitive impairment could be related to the disruption of the cerebro-ponto/reticulo-cerebellar-thalamocortical loops caused by the broken communication between the default-mode network and cerebellum [62].

Another interesting observation in our study is the laterality of ECT-modulated FNC neuroplasticity. Changes in FNC between the right cerebellum and PCC and between the cerebellum and right IPL are associated with cognitive and antidepressant outcomes, respectively. The lateralization of brain changes has been documented in ECT literature [24, 50, 63, 64], although its underlying mechanisms are still unclear. Considering the right laterality of the electric field [24, 29], our findings might suggest a potential linkage between electric field laterality and functional connectivity laterality. Numerous studies believe that the laterality of brain changes might be due to the predominant side of stimulation [50, 65]. We speculate that the E-field-related FNC lateralization might also be associated with the major depressive episodes themselves since functional brain abnormalities in the right hemisphere have been widely reported in the mood- and stress-related disorders [64].

Our investigation further demonstrated that cerebro-cerebellar FNC is a potential mediator between the E-field and clinical outcomes. Increased E-field strength is associated with decreased FNC between the cerebellum and PCC, which results in cognitive impairment after ECT. Meanwhile, increased E-field strength is associated with decreased connectivity between the cerebellum and right IPL, which results in improved antidepressant outcomes. These mediation effects show similar and unique patterns compared with previous investigations that focused on structural neuroplasticity [24, 29]. On one hand, both functional and structural neuroplasticity show mediation effects on the association between E-field strength and ΔHDRS. On the other hand, while the link between structural neuroplasticity and cognitive impairment is less robust, functional neuroplasticity significantly mediates the effect of E-field strength on the DKEFS Letter Fluency Test score. These findings may suggest complementary but differential processes of functional and structural neuroplasticity [50]. Additional pre-clinical studies with advanced analytical methods such as data fusion are needed to elucidate the mechanistic link between electric field, functional neuroplasticity, structural neuroplasticity, and clinical outcomes.

There are several limitations in the present study that might influence the result interpretation. First, as an important therapeutic component of ECT, seizure activity might impact the effects of the electric field on functional neuroplasticity and clinical outcomes [66, 67]. Our investigation did not assess the potential relationships between seizure activity on functional connectivity changes and clinical outcomes. The possible role of seizures on functional neuroplasticity is still unclear. Future studies can employ similar mediation models with topographical ictal power to unveil the impact of E-field and ictal power on ECT’s therapeutic and iatrogenic effects. Second, our study included adult patients with MDD with or without psychotic features and two electrode placements (RUL or BT). Our sample size was underpowered for the investigation of the group- or electrode-specific changes in functional neuroplasticity. In future studies with larger sample sizes, we can assess the effects of psychosis and electrode placement on functional neuroplasticity. We can also confirm and probe the relationships between electric field, functional neuroplasticity, and clinical outcomes within each sub-group. Third, subjects recruited in our study discontinued antidepressant medications but did receive as-needed medications for anxiety and sleep. Larger samples with different medications will be needed to assess these complex relationships with both antidepressant and cognitive outcomes. Fourth, our present study only focused on the functional neuroplasticity that is related to the electric field and ECT responses. Existing work has suggested potential relationships between structural neuroplasticity, electric field, and clinical outcomes [24, 29]. Despite these findings, the relationship between structural and functional neuroplasticity in the context of ECT response remains unknown. Structural and functional neuroplasticity might play overlapping and complementary roles in antidepression-related circuitry. In future studies, advanced fusion analysis tools can be combined with mediation analysis to elucidate more details of the mechanisms underlying structural and functional neuroplasticity induced by ECT. Fifth, our work only focused on cerebro-cerebellar connectivity because we believed that the cerebro-cerebellar functional neuroplasticity might imply a potential neural pathway for the mitigation of ECT-induced side-effects and improvement of antidepressant outcomes. The present analyses can be extended to whole-brain connectivity in the future to draw a more comprehensive picture of E-field-related connectivity signatures for ECT outcomes. Finally, in the present study, only the letter fluency of the verbal fluency test is significantly correlated with the E_brain_. We did not identify a significant correlation between ∆DKEFS Verbal Fluency - Category Fluency and the E_brain_ (Results are provided in the supplementary materials). Although our results provide new evidence that E-field can differentially impact cognitive outcomes, future studies can focus on the E-field strength in the specific brain regions that have been linked to the ECT mechanism, which will help to capture better relationships between E-field, functional neuroplasticity, and cognitive outcomes.

In conclusion, this investigation provides further support for the construct of optimal ECT dosing in relation to the E-field. Increased E-field influences cerebellar-cerebral FNC and improves antidepressant outcomes. However, increased E-field also changes other cerebellar-cerebral FNC and adversely impacts cognitive performance. The relationship between E-field and cognitive performance may serve as an upper limit for determining ECT dosing. With pre-ECT imaging, an individual’s E-field may be calculated prior to the ECT series based on the given electrode placement. E-field informed ECT has the potential to eliminate the trial-and-error dosing strategy (RUL with BT contingency) that was utilized in this investigation. An individualized amplitude may be then calculated to optimize antidepressant outcomes and minimize cognitive risk.

## Supplementary information

Supplementary Materials

## Data Availability

The code of the Neuromark framework and the Neuromark template have been released and integrated into the group ICA Toolbox (GIFT, https://trendscenter.org/software/gift/), which can be downloaded and used directly by users worldwide. Other MATLAB codes of this study can be obtained from the corresponding author.
