# Supplementary material for: Cerebro-cerebellar functional neuroplasticity mediates the effect of electric field on electroconvulsive therapy outcomes
Source: Transl Psychiatry. 2023 Feb 6;13:43. doi: 10.1038/s41398-023-02312-w (PMC9902462; doi:10.1038/s41398-023-02312-w)
Supplement: Supplementary file 1 — Supplementary Materials [file 41398_2023_2312_MOESM1_ESM.docx]

Supplementary Materials for

“Cerebro-cerebellar Functional Neuroplasticity Mediates the Effect of Electric Field on Electroconvulsive Therapy Outcomes”

# Study Design

Each subject was randomly assigned to 600, 700, and 800 mA before the first ECT treatment and started the ECT series with the right unilateral (RUL) electrode placement. Subject randomization was completed with a 1:1:1 ratio for each study arm. Subjects received clinical, neuropsychological, and imaging assessments pre- (v1), mid- (after the sixth ECT treatments, v2), and post-ECT (within one week of finishing the ECT series, v3). Initially, 47 subjects received ultra-brief pulse width (0.3 ms). Our planned interim data analysis showed a trend toward the lower efficacy of the 600 mA arm and thus the remaining 15 subjects in the present study received brief pulse width (1 ms). The rationale for the increased pulse width, as approved by the National Institutes of Health and the study Data Safety Monitoring Board, was to improve the efficacy of the lower amplitude arm. Subjects were switched to bitemporal electrode (BT) placement with 800 mA and 1.0 ms pulse width if they showed a <25% reduction from baseline HDRS_24_ at the v2 session.

Individual seizure thresholds were determined by the first ECT session with subsequent treatments provided at six times the seizure threshold with similar adjustments to pulse train duration and frequency across arms ^1^. To ensure adequate seizure morphology and duration, we permitted further adjustments to charge based on the clinical judgment. The appropriate dose of methohexital, a general anesthetic, and succinylcholine, a depolarizing neuromuscular blocker was determined by the treating anesthesiologist.

# Clinical and Cognitive Assessments.

Blinded to treatment-arm assignment, trained raters performed the clinical and cognitive assessments at each visit (v1~v3). ECT Appropriateness Scale was included in the initial study visit to assess the indication for ECT ^2^. Maudsley Staging Method was used to measure the Treatment Resistance ^3^ and Framingham Stroke Risk Profile was used to measure vascular burden ^4^.

Hamilton Depression Rating Scale 24-item (HDRS_24_) total score was the primary measure of clinical outcomes of depression severity. Each visit included the Test of Premorbid Function (TOPF), which is an estimate of premorbid intellectual function. The Delis Kaplan Executive Function System (DKEFS) which measured processing speed, verbal fluency, inhibition, and cognitive flexibility was used in our study to evaluate the cognitive performance ^5^. Measures from the DKEFS included Verbal Fluency, Category Fluency, and Color-Word Interference, and the entire neuropsychological battery was evaluated with a completion time of less than one hour. Demographic information of the participants is provided in Table S1.

Demographics and clinical characteristics of participants

|  | Characteristics | |  | T statistic |
| --- | --- | --- | --- | --- |
|  | MDD (n=50) | v1 | v3 | p value |
| Age (years) | 65.54 ± 8.92 | n/a | n/a | n/a |
| Gender (female/male) | 35/15 | n/a | n/a | n/a |
| TOPF | 109.45 ± 11.61 | n/a | n/a | n/a |
| ECT treatments | 11±3.25 | n/a | n/a | n/a |
| Pulse width (0.3 ms/1 ms) | 21/29 | n/a | n/a | n/a |
| HDRS | n/a | 36.22±7.03 | 14.88±10.47 | 8.50 ×10^-18^ |
| DKEFS Verbal Fluency - Letter Fluency | n/a | 9.16±3.17 | 6.90±3.30 | 5.98×10^-7^ |

DKEFS = Delis Kaplan Executive Function System; ECT = Electroconvulsive Therapy; HDRS = Hamilton Depression Rating Scale; MDD = Major Depressive Disorder; TOPF = Test of Premorbid Functioning Score.

# Image Acquisition

3T-Siemens scanner acquired resting-state fMRI data with the following parameters: Repetition time (TR) = 745 ms, echo time (TE) = 29 ms, flip angle (FA) = 75°, slices = 192, voxel size = 2.0 × 2.0 × 2.0 millimeter (mm)^3^, and total acquisition time 4:58 (minutes:seconds). Each subject received one or two resting-state scans at each visit. T1 data was collected with the following parameters: TR = 2530 ms, TE = 1.64, 3.5, 5.36, 7.22, 9.08 ms, FA = 7.0°, slices = 192, field of view = 256, matrix 256 × 256, voxel size = 1.0 × 1.0 × 1.0 mm and total acquisition time 6:03. T2 data was collected with the following parameters: TR = 2530 ms, TE = 474 ms, FA = 120.0°, slices = 192, field of view = 256, matrix 256 × 256, voxel size = 1.0 × 1.0 × 1.0 mm and total acquisition time = 5:09.

# Image Preprocessing

We preprocessed the resting-state fMRI data using a combination of the FMRIB Software Library v6.0 (FSL) toolbox and Statistical Parametric Mapping 12 (SPM 12) toolbox, under the MATLAB 2019b environment. Volumes acquired with phase encoding in the anterior-posterior (AP) direction and volumes with phase encoding in the posterior-anterior (PA) direction were used with the FSL tool *topup* ^6,7^ to estimate the susceptibility-induced off-resonance field. The output field map coefficients were used to correct the distortion in the fMRI volume using the FSL tool *applytopup*. After distortion correction, we discarded 10 initial scans with large signal changes to allow the tissue to reach a steady state of radiofrequency excitation. Next, we performed a slice-timing correction to account for the timing difference in slice acquisition and then a rigid body motion correction to correct the head motion. The fMRI data were subsequently warped into the standard Montreal Neurological Institute (MNI) space using an echo-planar imaging (EPI) template and were slightly resampled to 3 × 3 ×3 mm^3^ isotropic voxels. The resampled fMRI images were further smoothed using a Gaussian kernel with a full width at half maximum (FWHM) = 6 mm.

# Comparing Individual Masks and Group Mask for fMRI Subject Selection

Whether subjects’ scans have good normalization to the EPI template is important for the ICA and the calculation of FNC. A method based on the comparison of the individual mask and the group mask was used in our study for the subject selection. This method ensures we have a high-quality mask and fMRI data for the retaining subjects and its effectiveness has been proven in previous studies ^8–11^.

First, based on the first fMRI time volume, we calculated the individual mask for each scan by setting voxels that are greater than 90% of the whole brain mean to 1. Next, we computed a group mask by setting voxels that are included in more than 90% of the scans to 1. For each scan, we then calculated the spatial correlations between the group mask and the individual mask. The spatial correlations were calculated using voxels within the top 10 slices of the mask, within the bottom 10 slices of the mask, and within the whole mask, resulting in three correlation values for each scan. If a scan has correlations larger than 0.75 for the top 10 slices, larger than 0.55 for the bottom 10 slices, and larger than 0.8 for the whole mask, we assume that this scan passes the QC. Subjects with at least one good scan (passed the data QC) in both v1 and v3 sessions were included in the further analysis.

# Neuromark Framework

A set of robust network templates were used to extract comparable ICNs across sessions and subjects for the ECT dataset. These network priors were extracted via a unified ICA framework, called the Neuromark framework. Two healthy controls datasets, the human connectome project (HCP, 823 subjects after the subject selection) and the genomics superstruct project (GSP, 1005 subjects after the subject selection) were used for the construction of the network templates. We chose these two datasets because they have different temporal resolutions and are preprocessed by different preprocessing pipelines. We want to generate network templates that are consistent and reproducible across various conditions.

Group ICA with a model order of 100 was performed on the GSP and HCP datasets respectively, and the captured ICs from the two datasets were matched by evaluating the correlation between their group-level spatial maps. Those pairs are considered consistent and reproducible across GSP and HCP datasets if their spatial correlation ≥ 0.4. A correlation value ≥ 0.25 has been shown to represent a significant correspondence (p < 0.005, corrected) between components ^12^. Here we used a higher threshold because we would like to identify more reliable and consistent ICs. The reproducible ICs pairs were further evaluated by examining their peak activations and low-frequency fluctuations of their corresponding time courses (TCs). 53 pairs of ICs were identified as ICNs, arranged into seven functional domains based on their anatomical and functional prior knowledge. We used the less noisy ICNs captured from the GSP dataset (Note that there are 53 ICNs from HCP which have similar spatial patterns) as the network templates to back-reconstruct spatial maps and TCs for each scan of each subject from the ECT dataset. By using the Neuromark framework, the identified ICs are corresponding across sessions, scans, and subjects, leveraging the feasibility of comparing results before and after ECT. Flowchart of the Neuromark framework for capturing reliable networks from the ECT data is provided in Figure S1.


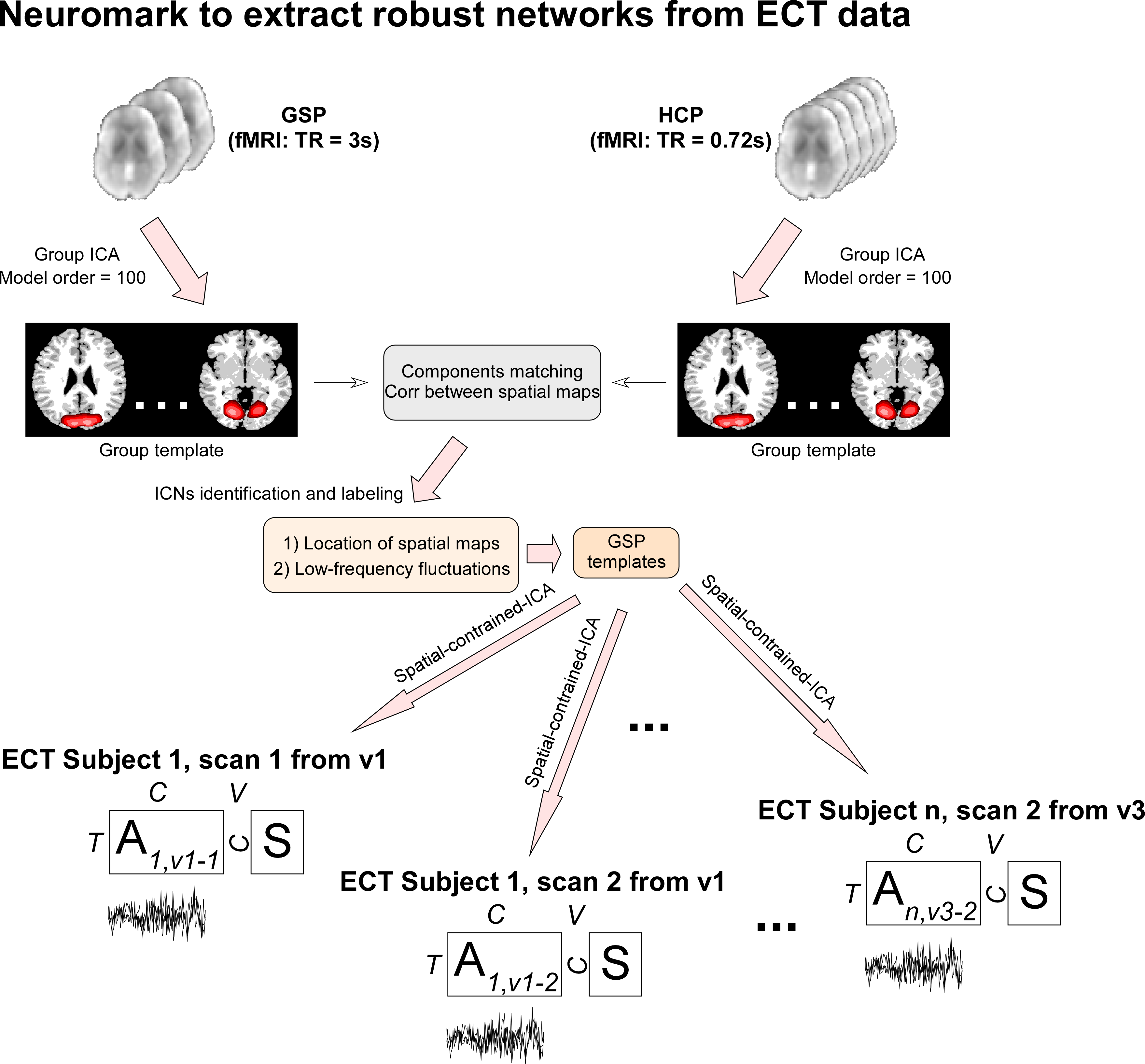


**Flowchart of the Neuromark framework to extract reliable networks and TCs from the ECT data.** Group ICA is performed on two independent healthy controls datasets and the estimated ICs are matched by the spatial correlation. Matched ICs are identified as ICNs according to their SMs and the ICNs are used as spatial templates to calculate spatial maps and TCs for ECT data.

# Intrinsic Connectivity Networks (Labeling, Coordinates, and Spatial Maps)

Peak Coordinates of Intrinsic Connectivity Networks (ICNs)

| **ICNs** | **X** | **Y** | **Z** |
| --- | --- | --- | --- |
| **Sub-cortical domain (SC)** | | | |
| Caudate (69) | 6.5 | 10.5 | 5.5 |
| Subthalamus/hypothalamus (53) | -2.5 | -13.5 | -1.5 |
| Putamen (98) | -26.5 | 1.5 | -0.5 |
| Caudate (99) | 21.5 | 10.5 | -3.5 |
| Thalamus (45) | -12.5 | -18.5 | 11.5 |
| **Auditory domain (AUD)** | | | |
| Superior temporal gyrus ([STG], 21) | 62.5 | -22.5 | 7.5 |
| Middle temporal gyrus ([MTG], 56) | -42.5 | -6.5 | 10.5 |
| **Sensorimotor domain (SM)** | | | |
| Postcentral gyrus ([PoCG], 3) | 56.5 | -4.5 | 28.5 |
| Left postcentral gyrus ([L PoCG], 9) | -38.5 | -22.5 | 56.5 |
| Paracentral lobule ([ParaCL], 2) | 0.5 | -22.5 | 65.5 |
| Right postcentral gyrus ([R PoCG], 11) | 38.5 | -19.5 | 55.5 |
| Superior parietal lobule ([SPL], 27) | -18.5 | -43.5 | 65.5 |
| Paracentral lobule ([ParaCL], 54) | -18.5 | -9.5 | 56.5 |
| Precentral gyrus ([PreCG], 66) | -42.5 | -7.5 | 46.5 |
| Superior parietal lobule ([SPL], 80) | 20.5 | -63.5 | 58.5 |
| Postcentral gyrus ([PoCG], 72) | -47.5 | -27.5 | 43.5 |
| **Visual domain (VS)** | | | |
| Calcarine gyrus ([CalcarineG], 16) | -12.5 | -66.5 | 8.5 |
| Middle occipital gyrus ([MOG], 5) | -23.5 | -93.5 | -0.5 |
| Middle temporal gyrus ([MTG], 62) | 48.5 | -60.5 | 10.5 |
| Cuneus (15) | 15.5 | -91.5 | 22.5 |
| Right middle occipital gyrus ([R MOG], 12) | 38.5 | -73.5 | 6.5 |
| Fusiform gyrus (93) | 29.5 | -42.5 | -12.5 |
| Inferior occipital gyrus ([IOG], 20) | -36.5 | -76.5 | -4.5 |
| Lingual gyrus ([LingualG], 8) | -8.5 | -81.5 | -4.5 |
| Middle temporal gyrus ([MTG], 77) | -44.5 | -57.5 | -7.5 |
| **Cognitive-control domain (CC)** | | | |
| Inferior parietal lobule ([IPL], 68) | 45.5 | -61.5 | 43.5 |
| Insula (33) | -30.5 | 22.5 | -3.5 |
| Superior medial frontal gyrus ([SMFG], 43) | -0.5 | 50.5 | 29.5 |
| Inferior frontal gyrus ([IFG], 70) | -48.5 | 34.5 | -0.5 |
| Right inferior frontal gyrus ([R IFG], 61) | 53.5 | 22.5 | 13.5 |
| Middle frontal gyrus ([MiFG], 55) | -41.5 | 19.5 | 26.5 |
| Inferior parietal lobule ([IPL], 63) | -53.5 | -49.5 | 43.5 |
| Left inferior parietal lobue ([R IPL], 79) | 44.5 | -34.5 | 46.5 |
| Supplementary motor area ([SMA], 84) | -6.5 | 13.5 | 64.5 |
| Superior frontal gyrus ([SFG], 96) | -24.5 | 26.5 | 49.5 |
| Middle frontal gyrus ([MiFG], 88) | 30.5 | 41.5 | 28.5 |
| Hippocampus ([HiPP], 48) | 23.5 | -9.5 | -16.5 |
| Left inferior parietal lobue ([L IPL], 81) | 47.5 | 5.5 | 22.5 |
| Middle cingulate cortex ([MCC], 37) | -15.5 | 20.5 | 37.5 |
| Inferior frontal gyrus ([IFG], 67) | 39.5 | 44.5 | -0.5 |
| Middle frontal gyrus ([MiFG], 38) | -26.5 | 47.5 | 5.5 |
| Hippocampus ([HiPP], 83) | -24.5 | -36.5 | 1.5 |
| **Default-mode domain (DM)** | | | |
| Precuneus (32) | -8.5 | -66.5 | 35.5 |
| Precuneus (40) | -12.5 | -54.5 | 14.5 |
| Anterior cingulate cortex ([ACC], 23) | -2.5 | 35.5 | 2.5 |
| Posterior cingulate cortex ([PCC], 71) | -5.5 | -28.5 | 26.5 |
| Anterior cingulate cortex ([ACC], 17) | -9.5 | 46.5 | -10.5 |
| Precuneus (51) | -0.5 | -48.5 | 49.5 |
| Posterior cingulate cortex ([PCC], 94) | -2.5 | 54.5 | 31.5 |
| **Cerebellar domain (CB)** | | | |
| Cerebellum ([CB], 13) | -30.5 | -54.5 | -42.5 |
| Cerebellum ([CB], 18) | -32.5 | -79.5 | -37.5 |
| Cerebellum ([CB], 4) | 20.5 | -48.5 | -40.5 |
| Cerebellum ([CB], 7) | 30.5 | -63.5 | -40.5 |


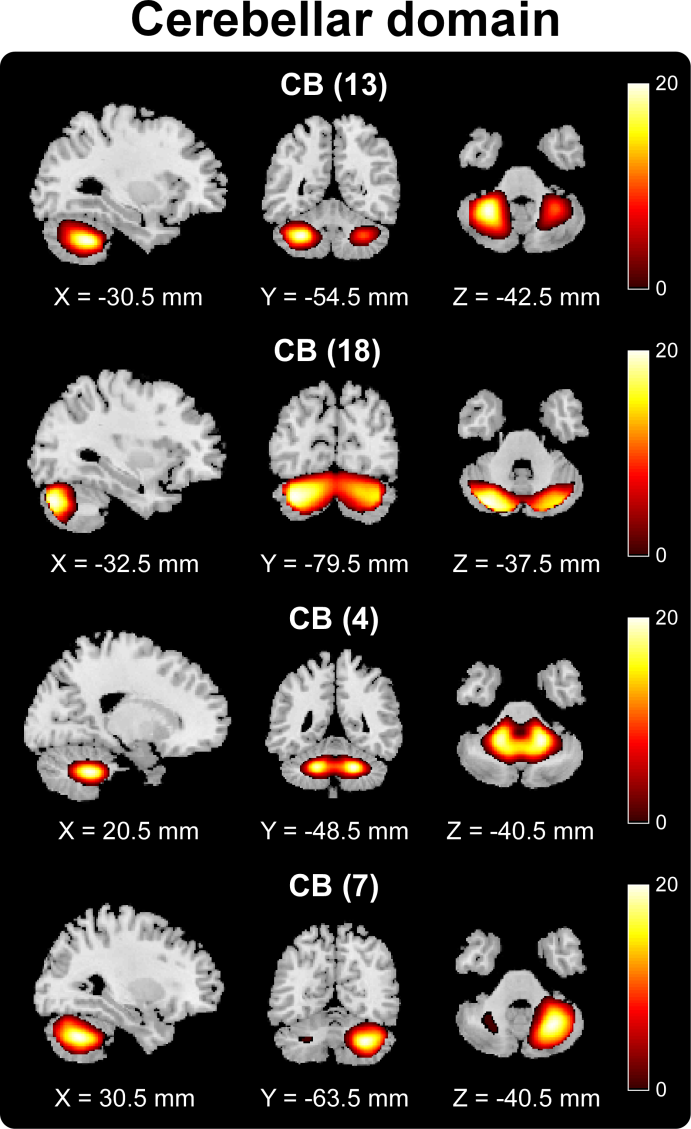


**Spatial maps of ICNs from cerebellar and cerebral domains.** ICNs are assigned into the one cerebellar domain and six cerebral domains shown in Figure 2 and are thresholded at |*t*|>10, where one-sample t-statistics have been computed across the single-subject spatial maps. Sagittal, coronal, and axial slices are shown at the maximal t-statistic for clusters larger than 3 cm^3^.


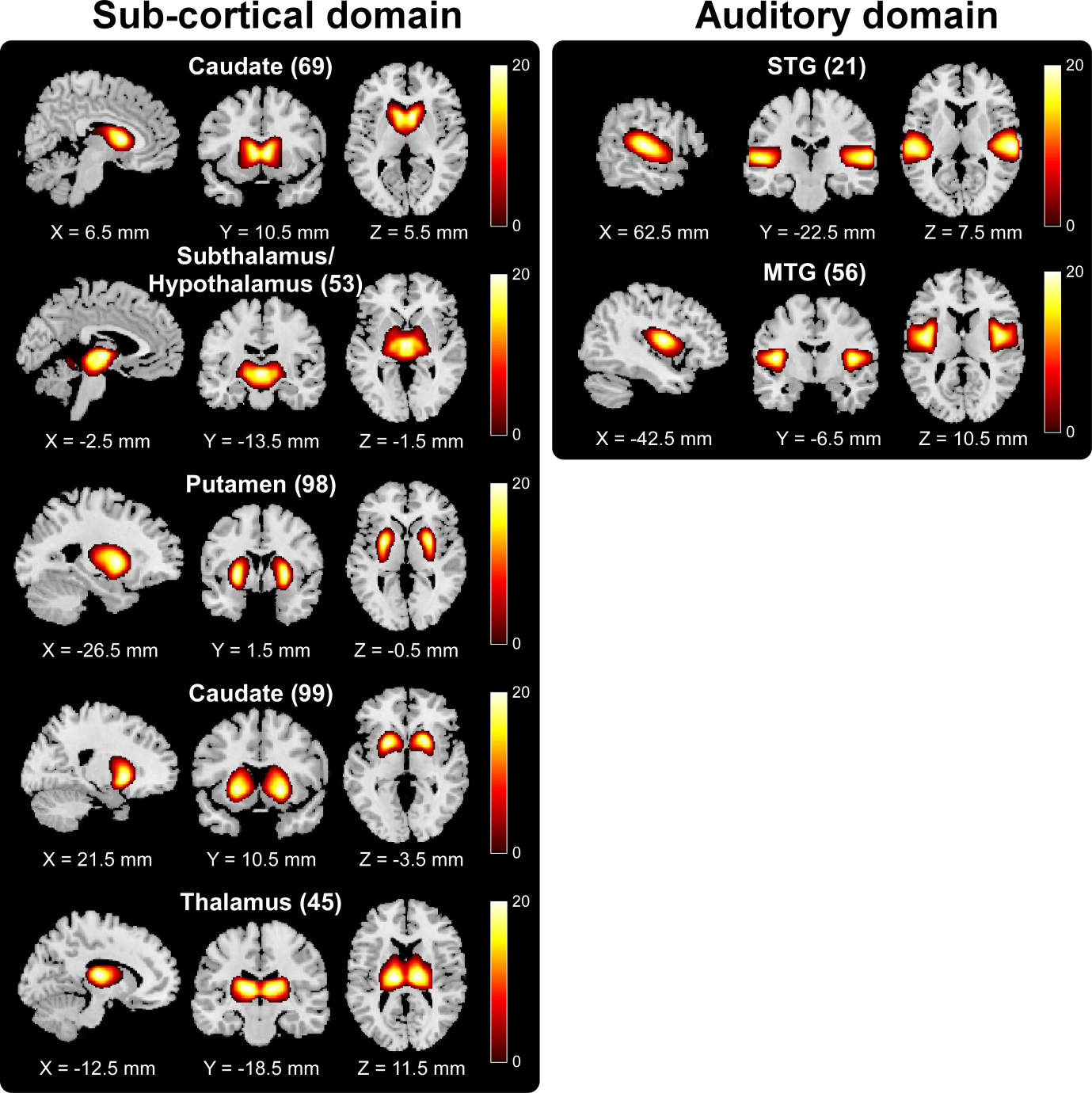

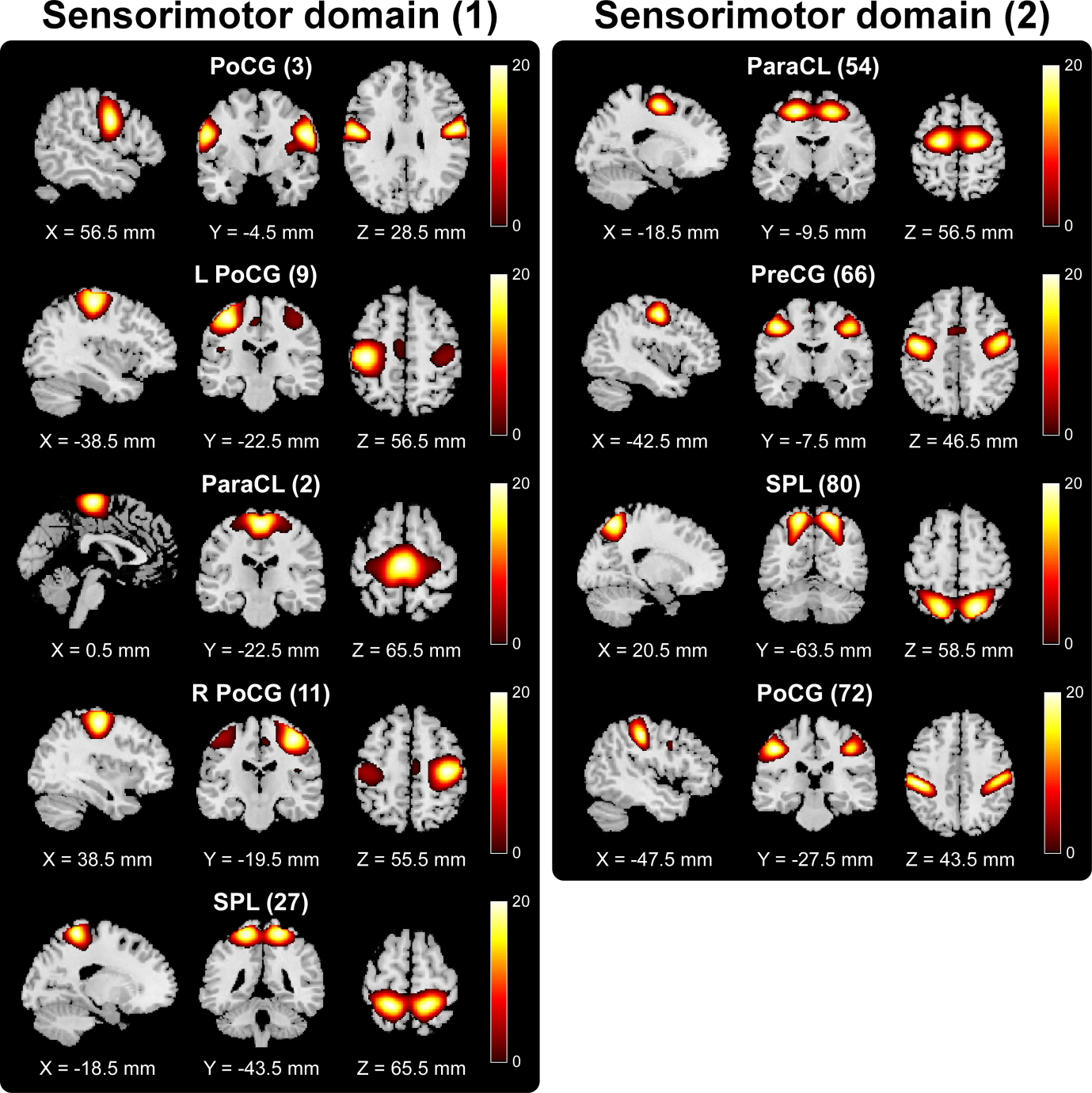

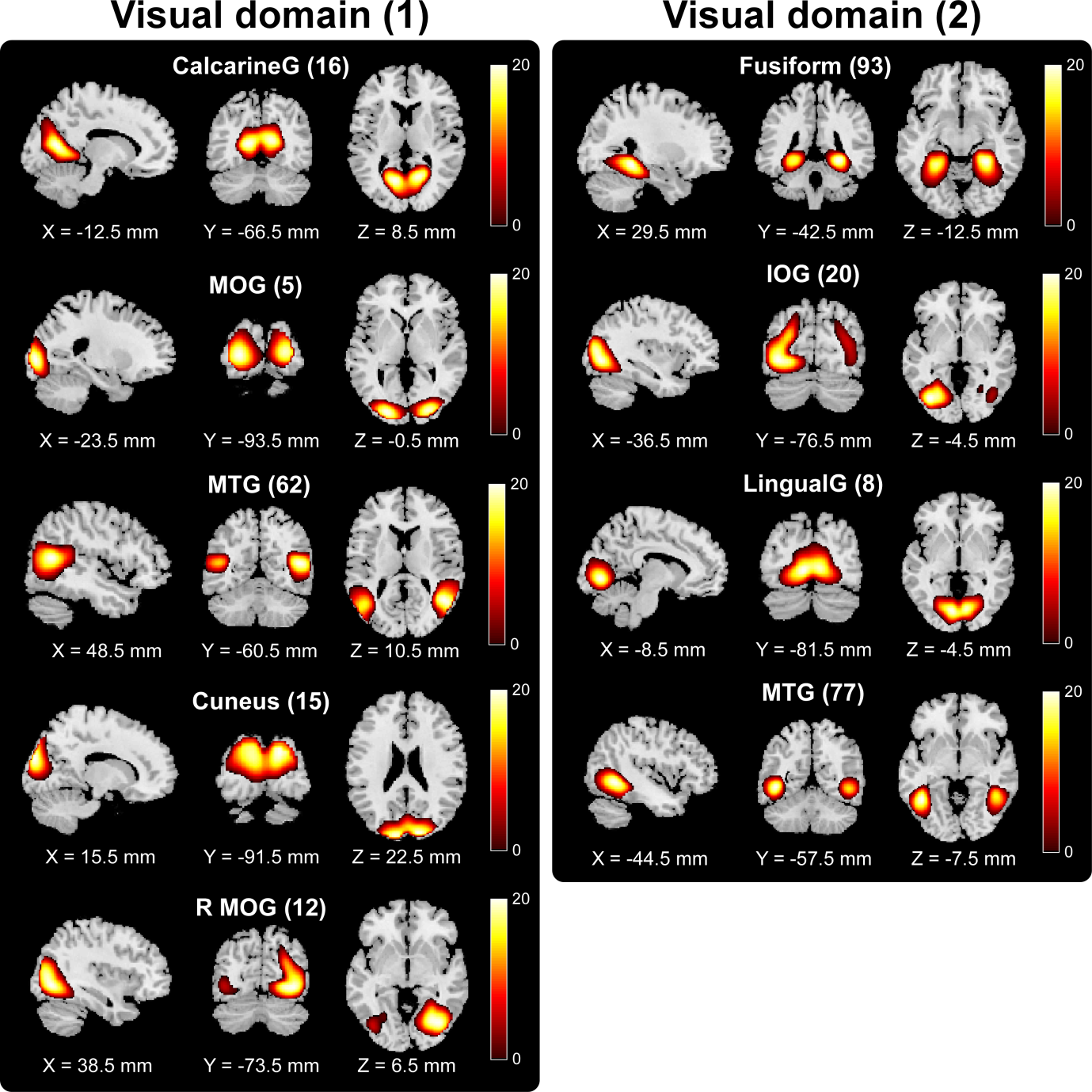

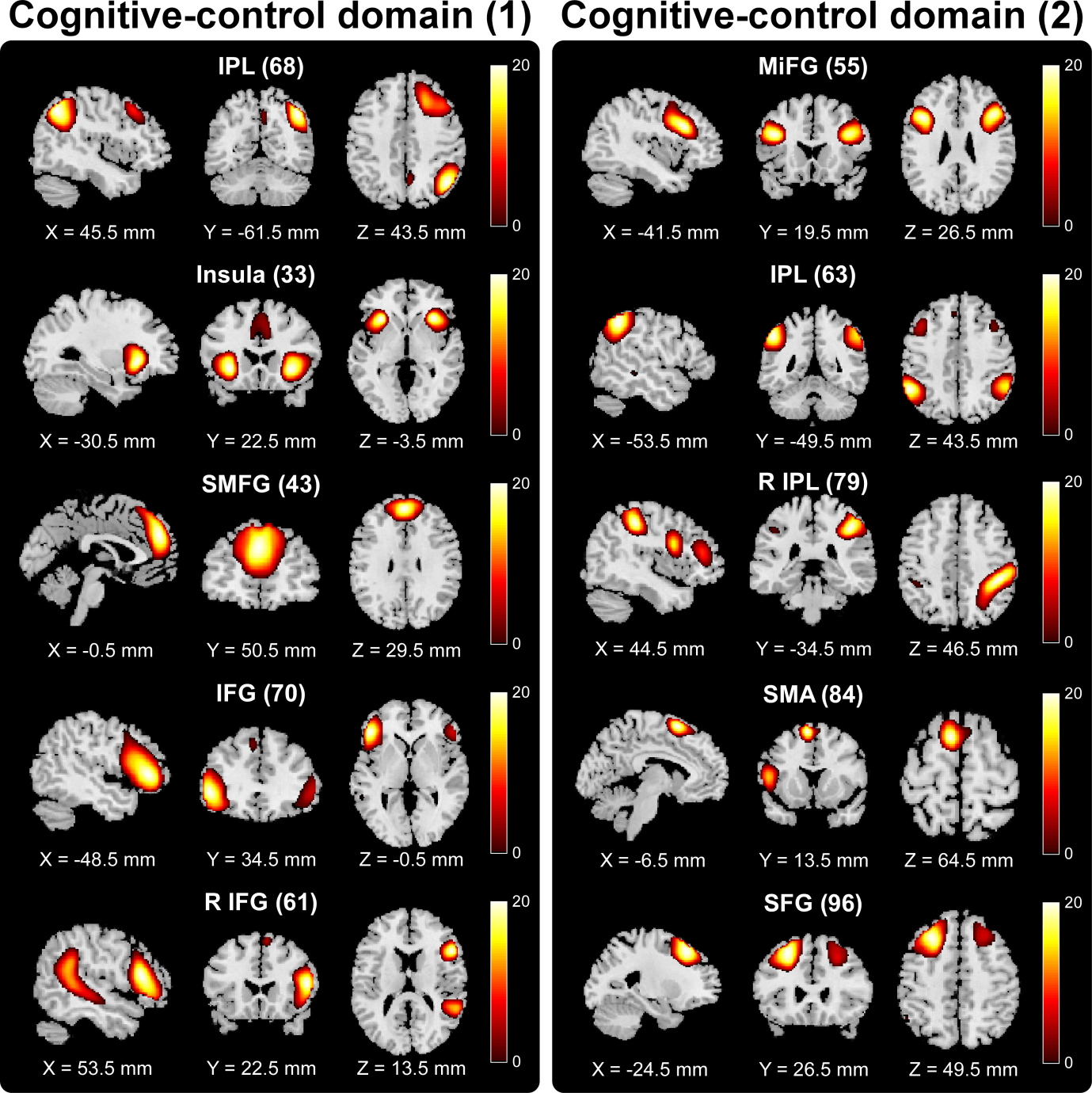

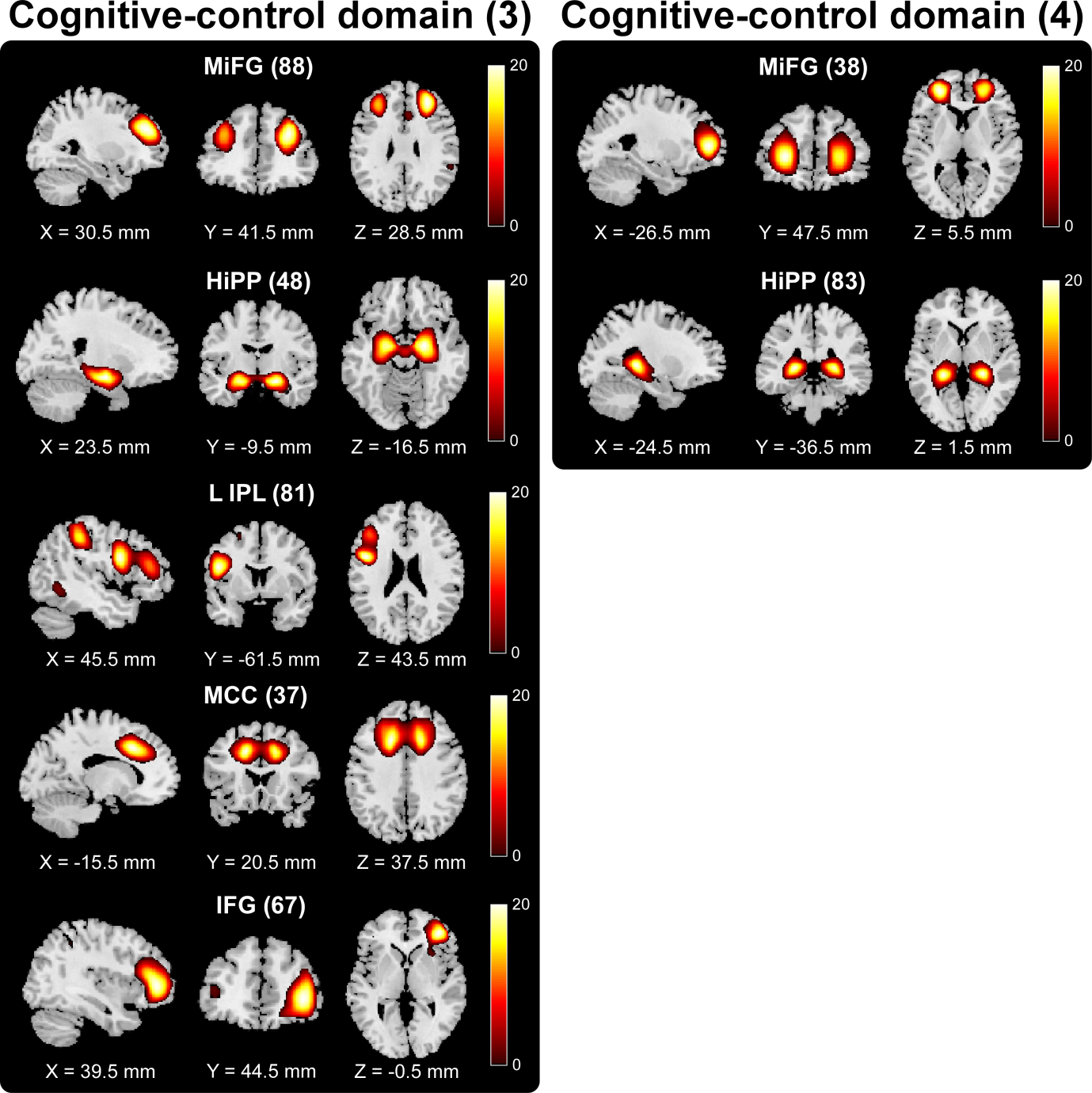

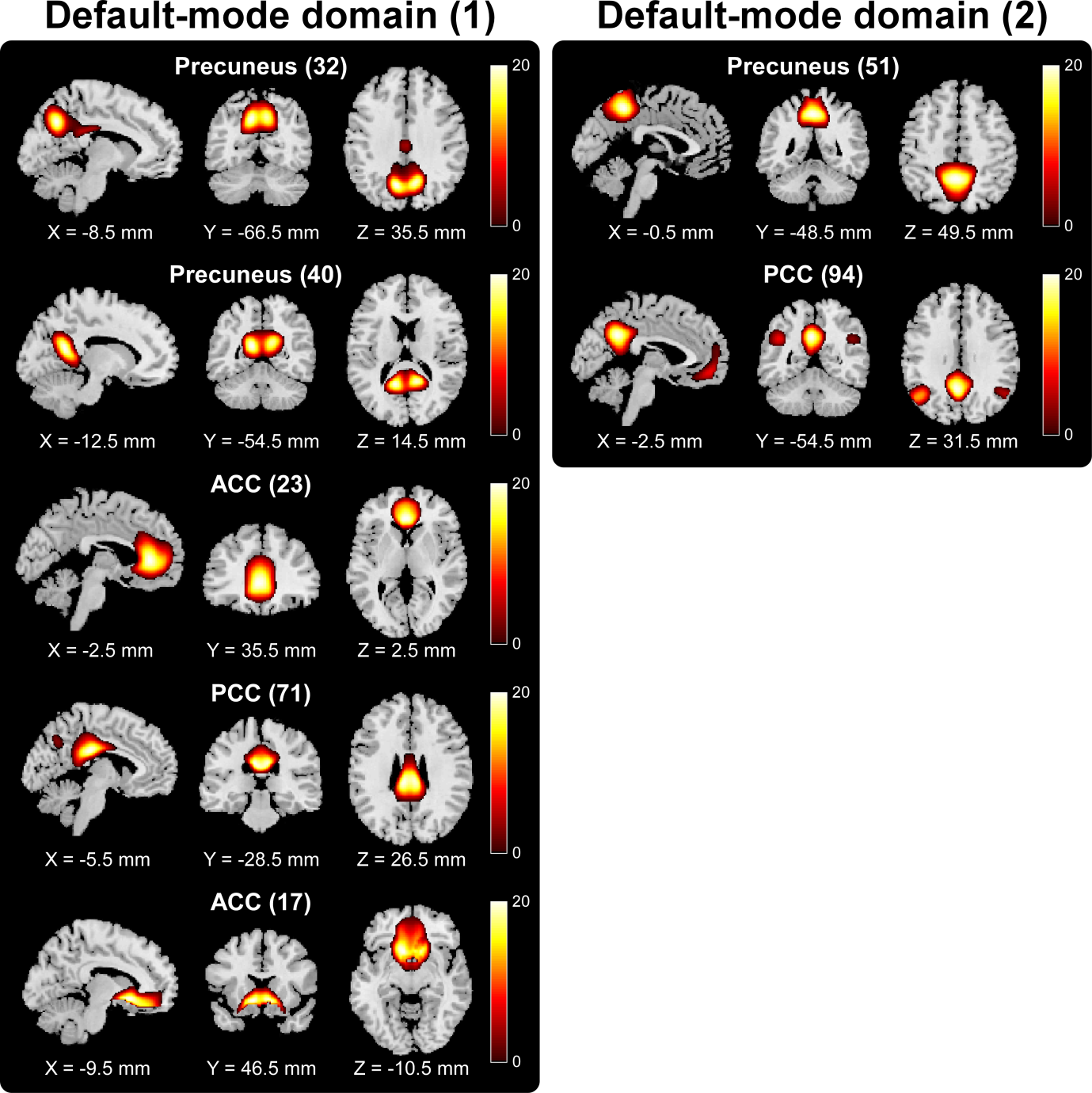


# Pulse Amplitude, FNC Neuroplasticity, and Clinical outcomes

In this study, E_brain_ was initially calculated at V/m at 1mA and then multiplied by the amplitude delivered. It should be noted that individuals have variable E_brain_ at 1mA due to anatomic differences in skull diameter, brain size, and skull thickness ^13^. Therefore, both anatomical information and amplitude will influence the E-field as well as the E_brain_. We did not include amplitude as one of the covariates because our work does not focus on either of the anatomical variability and ECT parameters separately but tries to combine their information in E-field modeling, to achieve the goal of individualized amplitude that might have a larger translational impact.

We performed additional experiments to explore the relationships between the amplitude (600, 700, and 800 mA), FNC neuroplasticity, and clinical outcomes. We performed the same analyses as in the main text, with the targeted measure E_brain_ replaced by the amplitude. We found that the amplitude does not correlate with the changes in DKEFS Verbal Fluency - Letter Fluency scaled score and HDRS (p = 0.1448 and p = 0.1503, respectively). Also, E_brain_-related FNC pairs are not correlated with amplitude. For example, the change of FNC between the cerebellum (4) and PCC is not correlated with the amplitude (p = 0.6495). Third, the mediation analysis shows that there is no mediation effect between amplitude and clinical outcomes through the FNC changes (e.g., all Path AB: p >0.05). The overall results suggest that E_brain_ has a higher explanatory value over stimulation amplitude by considering additional anatomical information to amplitude, which might have the potential to create a more standardized and consistent ECT dosing strategy to improve antidepressant and cognitive outcomes.

# E_brain_ related FNC Neuroplasticity Predict HDRS Changes (%)

Similar t-test analyses (as those in the section “*E_brain_-FNC neuroplasticity-cognition pathway*”) were performed for the HDRS in this section. We did not identify significant changes in FNC between the cerebellum and R_IPL (p > 0.05) when we used mean[E_brain_] as the threshold to divide subjects into two groups. However, we found that subjects with very small E_brain_ (< 75% × mean[E_brain_]) tend to have increased FNC between the cerebellum and R_IPL (p = 0.0094, t = -3.2029). In addition, unlike the results of cognitive impairment, significantly decreased HDRS after ECT can be observed in all samples (p = 8.50×10^-18^, t = 13.2939), in samples with decreased FNC between the cerebellum and R_IPL (p = 1.66×10^-12^, t = 12.8421), and in samples with increased FNC between the cerebellum and R_IPL (p = 2.15×10^-7^, t = 7.2651). This might be because 600 mA amplitude is still high enough to provide minor symptom improvements in MDD. Therefore, we performed an additional two-sample t-test to examine whether samples with different FNC changes (increased FNC or decreased FNC) have different levels of symptom improvement. We found that samples with increased FNC between cerebellum and R_IPL have smaller HDRS changes compared to the samples with decreased FNC (46.50% vs. 67.75%, p = 0.0131). The overall findings support the results of the mediation analysis, where lower E_brain_ is associated with decreased FNC, which might result in fewer antidepressant outcomes.

# FNC Neuroplasticity, E_brain_ and DKEFS Category Fluency

We performed the correlation analyses between FNC neuroplasticity, E_brain_ and the Category Fluency scaled score. Category Fluency is another important subdomain of the DKEFS test. Our result shows that there is no significant correlation between Category Fluency scaled score and E_brain_ (p = 0.3029, t = 1.0430, beta = 0.0273, effect size = 0.0346). Change of E_brain_-related FNC pairs did not correlate with change in DKEFS Verbal Fluency - Category Fluency (p > 0.05). Additionally, the mediation analysis shows that there is no mediation effect between E_brain_ and ∆DKEFS Verbal Fluency - Category Fluency through ∆FNC (Path AB: p > 0.05). For example, change of FNC between cerebellum (3) and MOG (one of the nine E_brain_-related FNC pairs) is not correlated with change in DKEFS Verbal Fluency - Category Fluency (p = 0.1244, t = -1.5680, beta = -2.9033, effect size = 0.0563). While the direct effect of E_brain_ on ∆DKEFS Verbal Fluency - Category Fluency is insignificant (Path C’: p > 0.05), the indirect effect through ∆cerebellum-MOG FNC is insignificant too (Path AB: p = 0.0667, Coeff = 0.0132, CI, 0.0086~0.0232). These results are in line with the previous work on structural neuroplasticity, where the E-field strength in the hippocampus had a significant relationship with Letter Fluency, not Category Fluency ^13^. Our present work and the previous work are based on different subsamples of the data (because of the differences in the quality control for functional and structural images) and different percentiles of E-field magnitudes (90% E_brain_ and 95% Hippocampal E-field strength), which further demonstrate the robustness of associations between E-field and Letter Fluency.

# References

1 Sackeim HA, Prudic J, Devanand DP, Nobler MS, Lisanby SH, Peyser S *et al.* A prospective, randomized, double-blind comparison of bilateral and right unilateral electroconvulsive therapy at different stimulus intensities. *Arch Gen Psychiatry* 2000; **57**: 425–434.

2 Kellner CH, Popeo DM, Pasculli RM, Briggs MC, Gamss S. Appropriateness for electroconvulsive therapy (ECT) can be assessed on a three-item scale. *Med Hypotheses* 2012; **79**: 204–206.

3 Fekadu A, Wooderson S, Donaldson C, Markopoulou K, Masterson B, Poon L *et al.* A multidimensional tool to quantify treatment resistance in depression: The Maudsley staging method. *J Clin Psychiatry* 2009; **70**: 177–184.

4 D’Agostino RB, Vasan RS, Pencina MJ, Wolf PA, Cobain M, Massaro JM *et al.* General cardiovascular risk profile for use in primary care: The Framingham heart study. *Circulation* 2008; **117**: 743–753.

5 Swanson J. The Delis-Kaplan Executive Function System:A Review. *Can J Sch Psychol* 2005; **20**: 117–128.

6 Andersson JLR, Skare S, Ashburner J. How to correct susceptibility distortions in spin-echo echo-planar images: Application to diffusion tensor imaging. *Neuroimage* 2003; **20**: 870–888.

7 Smith SM, Jenkinson M, Woolrich MW, Beckmann CF, Behrens TEJ, Johansen-Berg H *et al.* Advances in functional and structural MR image analysis and implementation as FSL. *Neuroimage* 2004; **23**: S208–S219.

8 Fu Z, Sui J, Espinoza R, Narr K, Qi S, Sendi MSE *et al.* Whole-brain Functional Connectivity Dynamics associated with Electroconvulsive Therapy Treatment Response. *Biol Psychiatry Cogn Neurosci Neuroimaging* 2021. doi:10.1016/j.bpsc.2021.07.004.

9 Du Y, Fu Z, Xing Y, Lin D, Pearlson G, Kochunov P *et al.* Evidence of shared and distinct functional and structural brain signatures in schizophrenia and autism spectrum disorder. *Commun Biol* 2021; **4**: 1–16.

10 Li K, Fu Z, Luo X, Zeng Q, Huang P, Zhang M *et al.* The Influence of Cerebral Small Vessel Disease on Static and Dynamic Functional Network Connectivity in Subjects along Alzheimer’s Disease Continuum. *Brain Connect* 2021; **11**: 189–200.

11 Fu Z, Sui J, Turner JA, Du Y, Assaf M, Pearlson GD *et al.* Dynamic functional network reconfiguration underlying the pathophysiology of schizophrenia and autism spectrum disorder. *Hum Brain Mapp* 2020; : hbm.25205.

12 Smith SM, Fox PT, Miller KL, Glahn DC, Fox PM, Mackay CE *et al.* Correspondence of the brain’s functional architecture during activation and rest. *Proc Natl Acad Sci U S A* 2009; **106**: 13040–13045.

13 Deng Z, Argyelan M, Miller J, Quinn D, … ML-M, 2021 U. Electroconvulsive therapy, electric field, neuroplasticity, and clinical outcomes. *Mol Psychiatry* 2022; **27**: 1676--1682.
